# Intralesional steroid injection for Mondor’s disease: A new approach based on a post-surgical case series

**DOI:** 10.5339/qmj.2025.2

**Published:** 2025-02-17

**Authors:** Wen-Tsao Ho

**Affiliations:** ^1^Department of Dermatology, Ho Wen Tsao Skin Clinic, New Taipei City, Taiwan *Correspondence: Wen-Tsao Ho. Email: derm.administration@gmail.com

To the Editor,

We have carefully reviewed the insightful case report by Avantifiori et al. entitled “Penile Mondor’s disease after open hernia repair surgery: A case report”, which was published in the Qatar Medical Journal.^1^ Their detailed exploration of penile Mondor’s disease (PMD) following inguinal hernia repair provides valuable perspectives on this rare condition.

In light of their work, we wish to present our findings from a series of cases of Mondor’s disease that occurred after axillary osmidrosis surgery. Our experience with this condition and the management strategies we used may contribute to a comparative viewpoint that enhances the broader understanding of Mondor’s disease.

Notably, in the present clinical practice, we have applied intralesional steroid injections for treating Mondor’s disease, which has not been previously reported. This treatment strategy is guided by dermatological practices for managing inflammation in conditions such as inflamed cysts or eczema.^2-3^ Given the efficacy observed in our cases, it may warrant further investigation as a potential therapeutic option.

## Case Series Summary

We identified Mondor’s disease in five patients (three cases with the affected left/right axilla, one case with the affected left axilla, and one case with the affected right axilla), following axillary osmidrosis surgery. The median age of the patients was 35 (31-45) years, with a median height of 158 (153-161) cm, a median weight of 54 (44-60) kg, and a median onset time of 2 (1-2) weeks. Diagnosis was based on clinical observations, including tenderness, palpable cords, pain, and occasionally visible bands. Management strategies varied, including conservative approaches for the right axilla and intralesional steroid injections for the left axilla. In particular, all patients received non-steroidal anti-inflammatory drugs (NSAIDs) as part of their treatment regimen.

## Discussion And Comparative Analysis

For abdominal Mondor’s disease, the use of NSAIDs combined with local infiltration of bupivacaine 0.25% resulted in significant improvement of symptoms.^4^ In this study, we identified Mondor’s disease in five patients following axillary osmidrosis surgery, with an incidence rate of 5 out of 500 surgeries (1%). Our findings indicate that intralesional steroid injections (median [range]: 5.5 [4-9] weeks) significantly reduced the time to symptom resolution in patients compared to those who received only conservative management (median [range]: 1.5 [1-3] weeks). The faster resolution of symptoms and reduced pain in patients who received steroid injections suggest that this approach may be beneficial in managing Mondor’s disease. This is in contrast to the approach reported by Avantifiori et al., who used low-molecular-weight heparin for the management of PMD, which resolved symptoms but resulted in residual venous ectasia.

In summary, by extending the previous dermatological practices with intralesional steroid injections for managing inflammatory conditions, our findings highlight the potential benefits of intralesional steroid injections for treating Mondor’s disease following surgery, especially in terms of reducing symptom duration and alleviating pain. This differs from other therapeutic options, such as anticoagulants, and provides a potentially more effective alternative in specific contexts. We hope that these insights will enhance the understanding of Mondor’s disease management and encourage further research into optimal treatment strategies.

### Competing interests

The author has no relevant financial or non-financial interests to disclose.
